# Process optimisation for improved chitinase production from marine isolate *Bacillus haynesii* and bioethanol production with *Saccharomyces cerevisiae*

**DOI:** 10.1007/s42770-025-01644-7

**Published:** 2025-03-06

**Authors:** Vishnupriya Govindaraj, Dinesh Kumar Anandan, Se-Kwon Kim, Ritu Raval, Keyur Raval

**Affiliations:** 1 Department of Chemical Engineering, National Institute of Technology, Surathkal, Mangalore, 575025 Karnataka India; 2 Department of Marine Science & Convergence Engineering, College of Science & Technology, University Erica Campus, Hanyang, Ansan, 11558 Republic of Korea; 3 Department of Biotechnology, Manipal Institute of Technology, Manipal Academy of Higher Education, Manipal, 576104 Karnataka India

**Keywords:** *Bacillus haynesii*, Bioethanol, Chitinase, Plackett-Burman Design (PBD), *Saccharomyces cerevisiae*, Taguchi

## Abstract

In the quest for sustainable fuel sources, chitin-based biorefineries are gaining recognition as chitin is the second most abundant bioresource after cellulose. This approach not only provides an effective method for converting shell waste from seafood processing into valuable bioethanol but also helps in waste management. In this study, *Bacillus haynesii*, a marine isolate, was investigated and this is the first report on optimisation of process parameters for chitinase production from *Bacillus haynesii*. The One Factor at a Time (OFAT) method was used to optimize process parameters including inoculum age, inoculum size, temperature, pH, and filling volume, with colloidal chitin identified as the best carbon source for the growth of *Bacillus haynesii*. The Plackett-Burman Design (PBD) was employed to screen media components, followed by optimization using the Taguchi Orthogonal Array method. The media components investigated included glycerol, yeast extract, MnCl_2_·4H_2_O, MgSO_4_·7H2O, NH_4_Cl, and colloidal chitin. As a result, the optimized media—comprising 7.5 g/L yeast extract, 7.5% (w/v) glycerol, 0.6% (w/v) colloidal chitin, 1.44 g/L MnCl_2_·4H_2_O, and 1.2 g/L MgSO_4_·7H_2_O—yielded an enzyme activity of 6.85 U/mL with a specific activity of 28.87 U/mg. Furthermore, ethanol production from chitin oligosaccharides by *Saccharomyces cerevisiae* was quantified using the potassium dichromate oxidation method, achieving a bioethanol concentration of 2.48% v/v from 33.18 g/L of chitin oligosaccharides. These results demonstrate the potential of *Bacillus haynesii*-derived chitin oligosaccharides as a promising substrate for bioethanol production.

## Introduction

Despite fossil fuels being a foundational element of the world’s energy supply, their combustion releases greenhouse gases, exacerbating global warming. The gradual depletion of fossil fuel reserves has driven the search for alternative natural resources to meet the increasing energy demands of a growing global population [1]. Bioethanol, derived from renewable resources, has gained recognition as a promising alternative to reduce reliance on conventional fossil fuels. However, the choice of sustainable and environmentally friendly biomass is crucial to ensuring the ecological viability of bioethanol production [2]. Chitin-based biorefineries are attracting increasing attention for their ability to convert chitinous waste into biofuel. In the quest for innovative bioethanol production methods, chitin oligosaccharides derived from chitin waste have demonstrated significant potential as a promising feedstock [3].

In nature, chitin is the second most abundant polysaccharide after cellulose [4]. It is composed of N-acetylglucosamine residues linked by β-1,4-glycosidic bonds [5]. This readily available bioresource is a major structural component of arthropods, crustaceans, mollusks, insects, and fungal cell walls [6]. These organisms contribute approximately 100 billion tonnes of chitin annually [7]. Waste management is a significant challenge for seafood processing industries, which generate 6–8 million tons of shell waste, with India alone producing up to 80,000 metric tons. This waste is often either dumped into the ocean or disposed of in landfills, posing a major environmental concern [8, 9]. Utilizing this chitin bioresource to generate fuel is an effective strategy, though the insolubility and crystalline nature of chitin limit its industrial applications [10].

Chitin oligosaccharides, derivatives of chitin obtained through chemical or enzymatic methods, possess better solubility and hold potential industrial applications. Chemical methods involve harmful chemicals that result in environmental pollution and complex products, increasing purification costs. The non-toxic, environmentally friendly enzymatic method uses chitinase, leading to the specific production of desired products [11, 12].

Chitinases, which belong to glycoside hydrolase families 18, 19, or 48, hydrolyze chitin into N-acetylglucosamine and its oligomers. Based on their mode of action, chitinases are classified as endo-chitinases (E.C 3.2.1.14) and exo-chitinases (E.C 3.2.1.30). Endo-chitinase cleaves internal sites, generating low molecular weight multimers such as diacetylchitobiose, chitotriose, and chitotetrose. Exochitinases are further categorized into chitobiosidases (E.C 3.2.1.29) and β-N-acetylglucosaminidases (E.C3.2.1.52), which act on chitin from the non-reducing end, resulting in diacetyl chitobiose and oligomers that generate N-acetylglucosamine monomers, respectively [13]. Sources of chitinases include bacteria, fungi, nematodes, insects, and plants [14, 15].

Several studies have identified and characterized chitinase from marine resources such as *Bacillus aryabattai* [10], *Paenibacillus* sp. AD [16], *Microbulbifer* sp. BN3 [17], *Beauveria bassiana* [18], *Vibrio rotiferianus*, and *Vibrio harvey* [19]. Various processes have been developed for chitinase production but optimizing physical and chemical parameters such as incubation temperature, pH, and inoculum size is crucial for improving microorganism growth [20]. Several authors have reported the optimization of process parameters for chitinase production using the One Factor at a Time (OFAT) approach [6, 21–23]. However, this process is time-consuming and involves numerous experimental runs, making it difficult to study the combined effects of process parameters on chitinase production [24]. Statistical optimization is preferred to screen many factors in less time, allowing the study of each factor’s effect and interactions on response. Chitinase production and optimization using Response Surface Methodology (RSM) have been widely documented [25–28]. Full factorial design allows for the simultaneous evaluation of multiple factors at discrete levels by analyzing all possible combinations. However, this approach demands an extensive number of experimental runs, increasing the complexity and resource requirements. To mitigate these challenges, the Taguchi method provides a statistically efficient alternative [29], By employing orthogonal arrays, the Taguchi approach enables the systematic investigation of multiple factors across the full parameter space with a significantly reduced number of experimental trials. Several studies have demonstrated the successful application of the Taguchi method in optimizing chitinase production. This approach uses an orthogonal array to study many factors across the entire parameter space with the least number of experimental runs. Some authors have explored the optimization of chitinase production using the Taguchi approach [30–32].

The primary objective of the present study was to optimize process parameters for chitinase production by *Bacillus haynesii* using the Taguchi approach. Additionally, the study explored ethanol production from chitin oligosaccharides using *Saccharomyces cerevisiae*. The present study provides valuable insights into the optimization of media components for an enhanced chitinase yield and productivity.

## Materials and methods

### Chemicals

The following chemicals were purchased from Hi-media, India; agar (GRM026); chitin flakes from shrimp shells (GRM1356); chitin powder (GRM10909); phenol crystal (AS022); sulphuric acid (AS015); NaCl (MB023); KCl (MB043); CaCl_2_.2H_2_O (MB034); MgCl_2_.6H_2_O (MB040); MgSO_4_.7H_2_O (GRM683); M9 minimal medium salts (5X) (G013); Tris-Cl (ML013); 3,5-dinitro salicylic acid (DNSA), A.G (GRM-1582); glycerol (MB060), bovine serum albumin (MB083), Bbadford reagent (ML106), potato dextrose agar (MH096).

### Culture conditions for *Bacillus haynesii*

In our previous study, screening, isolation, biochemical and molecular characterisation of marine isolate *Bacillus haynesii* was explored [1]. Colloidal chitin being a substrate for chitinase production was prepared according to the protocol described by Murthy et al., 2012 [33]. Further, chitin content in colloidal chitin was quantified [34]. Petri plates containing M9 minimal media supplemented with 1% colloidal chitin and 1.5% agar wasincubated at 37 °C for 48 h. The media was prepared using Artificial Sea water (0.99 g/L MgSO_4_.7H_2_O, 0.25 g/L CaCl_2_, 1.5 g/L MgCl_2_.6H_2_O, 26.29 g/L NaCl, 0.74 g/L KCl). The isolated single colonies of *Bacillus haynesii* were inoculated in a pre-inoculum media and incubated for 24 h, 150 rpm at 37 °C in shaker incubator. Pre-inoculum media (5%) containing the isolate was inoculated in the production media. The production media comprised of M9 minimal media with 12% (w/v) glycerol, 2 g/L yeast extract, 1% w/v colloidal chitin and artificial sea water.

### Protein quantification

Protein concentration was determined using Bradford assay [35]. The reaction mixture contained 1600 µL of Bradford reagent and 400 µL of the broth supernatant. The reaction mixture was vortexed and incubated in dark at room temperature for 5 min. BSA was used as a standard and absorbance was measured at 595 nm using UV-Visible Spectrophotometer (Thermofisher Scientific, MA, USA).

### Chitinase assay

Chitinase assay was estimated based on the concentration of reducing sugar released after the enzymatic hydrolysis. The reaction mixture, consisting of 1% colloidal chitin substrate and enzyme (broth supernatant) in a 1:1 ratio, was incubated with 1 mL of Tris-Cl buffer (10 mM, pH 6) at 37 °C for 1 h. The same procedure was followed for both the substrate and enzyme blanks. After incubation, the reaction mixture was centrifuged at 10,000 rpm for 10 min. To the obtained supernatant, 1mL of DNSA solution was added and incubated at 95 °C for 10–15 min. Absorbance was measured at 540 nm using UV-Visible spectrophotometer (Thermofisher Scientific, MA, USA). A standard curve was constructed using N-acetylglucosamine (NAG) at concentrations ranging from 2 to 10 mM, and the released NAG concentration was calculated using Eq. (1). One unit of chitinase activity is defined as the quantity of the enzyme required to release one micromole of NAG per minute under standard assay conditions [36].

1 $$Concentration\,of\,NAG\left( {mM} \right) = \frac{{Absorbance\,at\,540\,nm}}{{0.64}}$$

### Enhancement of chitinase production using the one-factor-at-a-Time (OFAT) approach

#### Effect of carbon sources

The effect of different carbon sources on chitinase production was evaluated by supplementing M9 medium with 0.2% of various mono-, di-, and polysaccharides in Erlenmeyer flasks. The media were inoculated with 5% (v/v) of a 24-h starter culture and incubated at 37 °C for 48 h with continuous shaking at 150 rpm.

#### Effect of temperature

The optimum temperature for chitinase production was determined by inoculating 5% (v/v) of a 24-h pre-inoculum into M9 medium supplemented with 1% colloidal chitin. Cultures were incubated at temperatures ranging from 25 °C to 50 °C for 48 h at 150 rpm. The experiments were performed in duplicate using Erlenmeyer flasks, and chitinase activity was measured using the DNSA method.

#### Effect of pH

The initial pH of the M9 medium containing 1% colloidal chitin was adjusted to values ranging from 3 to 11 in separate Erlenmeyer flasks. The experiment was conducted at the optimized temperature with agitation at 150 rpm, and chitinase activity was measured using the DNSA method.

#### Effect of inoculum age and inoculum percentage

Erlenmeyer flasks containing 10 mL of M9 medium supplemented with 1% colloidal chitin were inoculated with a 24 h pre-culture. The inoculum percentage was optimized within a range of 1–20%, while the effect of inoculum age was evaluated at 24, 48, 60, 72, and 90 h. Post-inoculation, the flasks were incubated at 37 °C with agitation at 150 rpm. Samples were collected at regular intervals, and chitinase activity was assessed using the DNSA assay.

#### Effect of culture volume on chitinase production

Sterile media were added to 100 mL Erlenmeyer flasks at different fill volumes of 5% (v/v), 7.5% (v/v), 10% (v/v), 15% (v/v), and 20% (v/v). The media were inoculated with a 24 h pre-inoculum and incubated at 37 °C with shaking at 150 rpm. Samples were collected at various time intervals, and chitinase activity was quantified using the DNSA assay.

### Statistical analysis

#### Experimental design and optimisation

Chitinase enzyme production from *Bacillus haynesii* was enhanced by identifying significant media components through Plackett-Burman design (PBD), followed by statistical optimization using the Taguchi approach.

#### Screening of significant media components by Plackett Burman design

Eleven independent variables were evaluated using the Plackett-Burman design to identify significant media components influencing *Bacillus haynesii* chitinase production. The experimental design and data analysis were performed using MINITAB statistical software (Version 21). The variables included in the design are listed in Table 1. The design generated 15 experimental runs, with each variable tested at two levels: high (+ 1) and low (-1). Experiments were performed in triplicates and the variables were examined by first order model represented in Eq. (2). Further, factors exhibiting significant effect on *Bacillus haynesii* chitinase production were optimised using Taguchi approach.

**Table 1:** Independent variables with corresponding levels in Plackett-Burman design

Factor	Name	Units	Minimum	Maximum
A	CaSO_4_	g/L	0.00	3.00
B	MgSO_4_. 7H_2_O	g/L	0.00	0.60
C	K_2_HPO_4_	g/L	0.00	6.00
D	KH_2_PO_4_	g/L	0.00	12.00
E	NaCl	g/L	0.00	10.00
F	MnCl_2_	g/L	0.00	0.12
G	(NH_4_)_2_SO_4_	g/L	0.00	2.00
H	Na_2_HPO_4_	g/L	0.00	14.00
J	NH_4_Cl	g/L	0.00	2.00
K	FeSO_4_	g/L	0.00	0.06
L	ZnSO_4_	g/L	0.00	0.06

2 $$Y = {\beta _0} + \sum {{\beta _i}{X_i}}$$

Where, Y is the response for chitinase production, β_0_ is the model intercept, β_i_ is the linear coefficient X_i_ is the level of independent variable.

#### Optimization of chitinase production using the Taguchi orthogonal array approach

Based on the PBD screening, four key factors affecting chitinase production, along with colloidal chitin, glycerol, and yeast extract, were selected for further optimization using the Taguchi approach. A 5-level array with a 6-factorial design was employed. The experiments were designed and analysed using 25 runs based on the L25 Orthogonal Array in MINITAB statistical software. Each experiment was performed in duplicate, and chitinase activity was measured using the DNSA method. Significant variables were identified based on a p-value of less than 0.05. Table 2 presents the Taguchi experimental design. To maximize chitinase production, the “bigger-the-better” criterion was applied, aligning with the objective of achieving the highest possible chitinase yield.

**Table 2:** L_25_ orthogonal array design for production of *Bacillus haynesii* chitinase

Run	Yeast extract	Glycerol	Colloidal chitin	MnCl_2_. 4H_2_O	MgSO_4_. 7H_2_O	NH_4_Cl	Response (enzyme activity)
	g/L	% (w/v)	% (w/v)	g/L	g/L	g/L	mU/mL
1	0	0	0	0	0	0	125
2	2.5	0	0.6	0.24	1.8	5	271
3	7.5	5	0	0.36	0.6	5	1174
4	0	10	2.4	0.48	2.4	5	935
5	5	10	0.6	0.36	0	2.5	1153
6	2.5	7.5	2.4	0	0.6	2.5	547
7	10	2.5	0	0.48	1.8	2.5	862
8	7.5	10	1.2	0	1.8	1.25	883
9	7.5	0	1.8	0.12	2.4	2.5	213
10	0	5	1.2	0.24	1.2	2.5	319
11	5	7.5	0	0.24	2.4	1.25	756
12	10	7.5	1.2	0.12	0	5	1056
13	10	0	2.4	0.36	1.2	1.25	321
14	0	2.5	0.6	0.12	0.6	1.25	297
15	10	10	1.8	0.24	0.6	0	707
16	0	7.5	1.8	0.36	1.8	3.75	577
17	5	2.5	1.8	0	1.2	5	1000
18	2.5	5	1.8	0.48	0	1.25	664
19	7.5	7.5	0.6	0.48	1.2	0	6361
20	5	0	1.2	0.48	0.6	3.75	418
21	10	5	0.6	0	2.4	3.75	377
22	5	5	2.4	0.12	1.8	0	1220
23	2.5	10	0	0.12	1.2	3.75	730
24	2.5	2.5	1.2	0.36	2.4	0	528
25	7.5	2.5	2.4	0.24	0	3.75	367

#### Ethanol production from chitin oligosaccharides using *Saccharomyces cerevisiae*

Ethanol production from chitin oligosaccharides using *Saccharomyces cerevisiae* was investigated as a fermentation process. *Saccharomyces cerevisiae* was inoculated into yeast extract-peptone-dextrose (YPD) medium, followed by fermentation to produce ethanol from chitin oligosaccharides. The inoculated media was incubated overnight at 30 °C, 160 rpm. Further, the cultured *Saccharomyces cerevisiae* cells were streaked on petri plates containing 2% of yeast extract-peptone-dextrose (YPD) agar and incubated at 30 °C until the isolated colonies were seen. Twenty millilitres of broth supernatant containing chitin oligosaccharides was inoculated with single isolated colonies of *S. cerevisiae*, and the culture was fermented at 30 °C for 48 h in batch mode. Five millilitres of the yeast culture were collected and centrifuged at 8000 rpm for 10 min. The supernatant containing ethanol was extracted using tri-n-butyl phosphate (TBP). Two millilitres of TBP were added to 2 millilitres of the standard ethanol solution and the sample. The mixture was vortexed for 1 min and allowed to stand undisturbed for 1 h. The upper layer, containing the ethanol-TBP complex, was then aliquoted into a separate test tube and subjected to a potassium dichromate oxidation assay.

#### Potassium dichromate oxidation assay

Ethanol estimation was carried out using the potassium dichromate oxidation assay. One milliliter of 10% dichromate reagent (prepared by dissolving 10 g of potassium dichromate in 100 mL of 5 M 98% concentrated sulfuric acid) was added to 1 mL of the extracted ethanol-TBP mixture and vortexed for 1 min. The mixture was allowed to separate into two layers. The bottom layer was collected and aliquoted into a separate test tube, and its absorbance was measured at 595 nm using a UV-Visible spectrophotometer (Thermofisher Scientific, MA, USA). A standard curve was constructed using ethanol at concentrations ranging from 1% (v/v) to 6% (v/v), and the ethanol concentration in the sample was calculated using Eq. (3).

## Results and discussion

### Effect of carbon sources

**Fig. 1:**
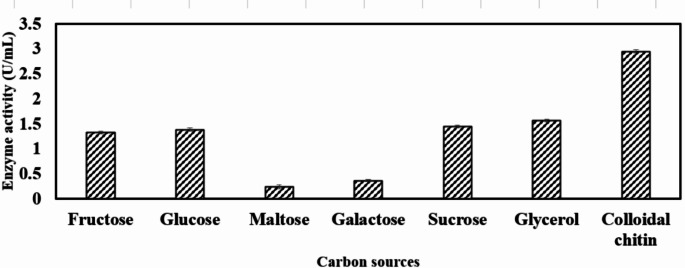
Effect of different carbon sources on *Bacillus haynesii* chitinase activity

Fructose, glucose, maltose, galactose, sucrose, glycerol, and colloidal chitin were evaluated to determine the most suitable carbon source for optimal chitinase production, as illustrated in Fig. 1. Among the seven carbon sources tested, colloidal chitin supported the highest chitinase activity of 2.85 U/mL when used as the substrate for *Bacillus haynesii* chitinase. Thus, colloidal chitin was identified as the preferred carbon source for *Bacillus* sp. WY22 [37] and *Bacillus* sp. R2 [38]. In a similar study conducted in *Bacillus cereus* GS02, the highest chitinase yield was observed with media supplemented with 1% colloidal chitin [20]. Similar results have also been reported by other groups [39–41].

Microbial chitinase can be induced by NAG, chitobiose, chitin and its oligomers [42]. In this study, colloidal chitin served as an inducer for chitinase production. Chitinase biosynthesis was comparatively lower in case of monosaccharides when compared to colloidal chitin. The minimum enzyme activity of 0.24 U/mL and 0.36 U/mL was observed in chitinase production medium supplemented with maltose and galactose respectively as the carbon source. Catabolite repression might be the reason behind inhibition of chitinase production in presence of monosaccharides. The results are in accordance with another study conducted on *Serratia marcescens* B4A [32]. Following colloidal chitin, glycerol was identified as an inducer for chitinase production, as it promotes increased cell biomass, resulting in a maximum chitinase activity of 1.5 U/mL. Consistent with these findings, glycerol has also been reported to enhance chitinase production in *Paenibacillus* sp. BISR-047 [42].

### Effect of temperature

**Fig. 2:**
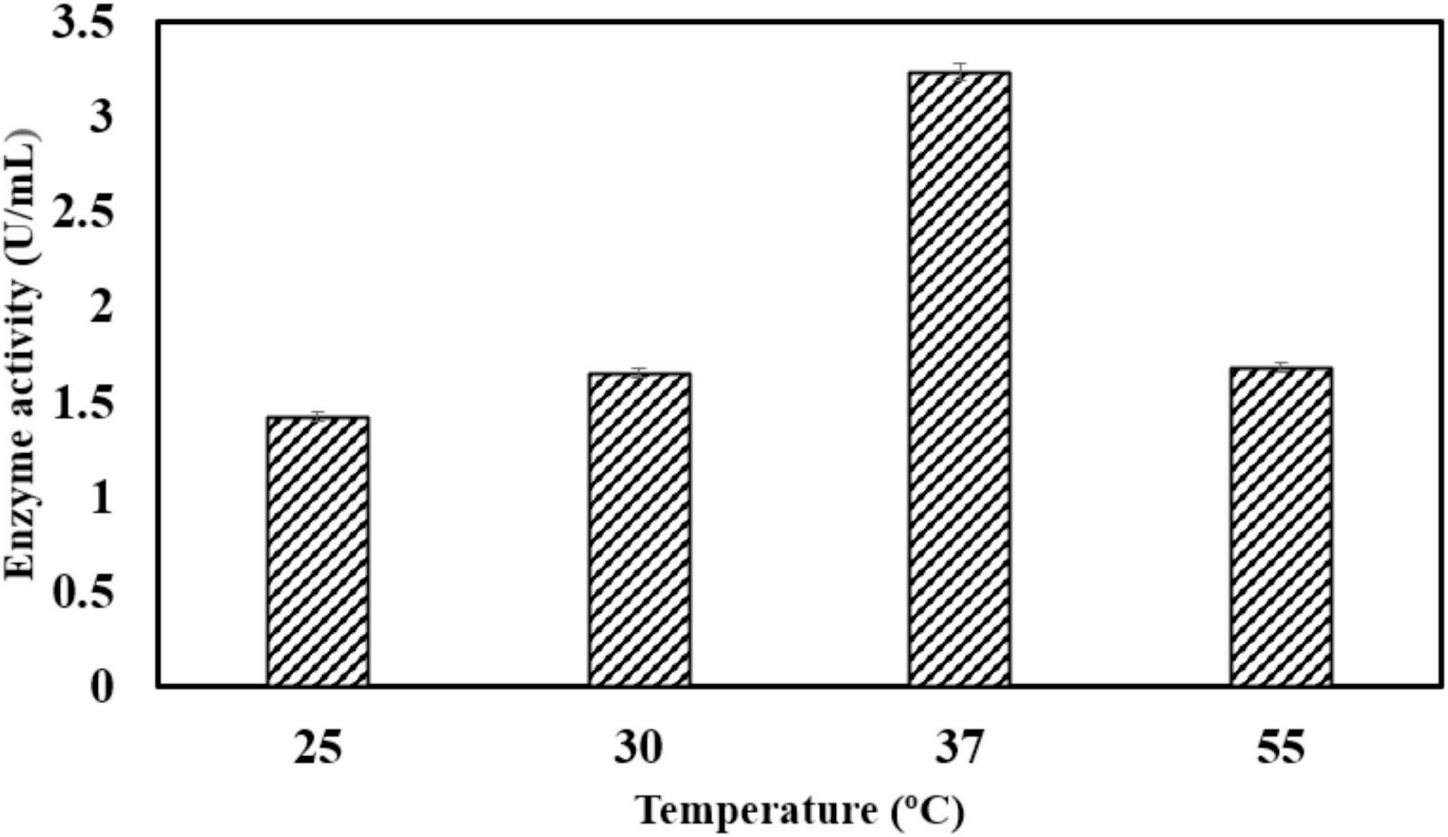
Effect of different temperature on *Bacillus haynesii* chitinase activity

Temperature of a culture medium has significant effects on protein denaturation, induction or repression of a metabolite, cell viability and cell death [43]. To analyse the optimum temperature for chitinase production, *Bacillus haynesii* was grown at different temperatures from 25 to 55 °C. The impact of incubation temperature on *Bacillus haynesii* chitinase production is represented in Fig. 2. The enzyme showed highest activity of 3.23 U/mL at 37 °C. The obtained results were in agreement with a study conducted on *Bacillus* sp [6]. and *Bacillus cereus* GS02 [20] where the optimum temperature was observed at 37 °C. *Bacillus thuringiensis* and *Bacillus licheniformis* produced chitinase at 40 °C [44]. In another study conducted on *Bacillus laterosporus* MML2270 the m was maximum chitinase production from was observed at 35 °C [45]. The optimum temperature of *Bacillus* sp. CH-2 isolated from fish waste dumping site was observed at 37 ºC [46]. In the present study an increase in temperature to 55 °C led to a reduction in the activity to 1.6 U/mL. Amino acids are critical for maintaining the three-dimensional structure of enzymes. The decline in enzyme activity at 55 °C can be attributed to increased molecular kinetic energy, which disrupts the interactions between amino acid residues. Consequently, the elevated temperature induced structural alterations in the enzyme, leading to reduced catalytic activity [47, 48].

### Effect of pH

**Fig. 3:**
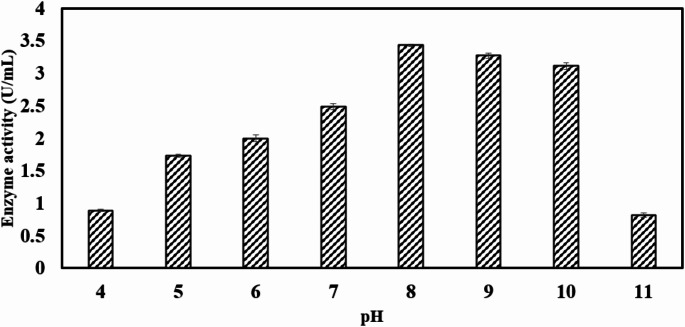
Effect of different pH on *Bacillus haynesii* chitinase activity

Growth and activity of the microbes are strongly influenced by medium pH as it regulates the availability of certain metabolic ions and permeability of bacterial membranes [43, 44]. Constant increase in enzyme activity was noted from pH range of 4–8 after which the chitinase activity was reduced. *Bacillus haynesii* exhibited maximum chitinase activity of 3.4 U/mL at pH 8 as represented in Fig. 3. *Bacillus haynesii* exhibited growth within a pH range of 6 to 10, while no survival was observed at pH levels below 4 or above 10. Variations in pH affect the ionization of functional groups in amino acid residues, which are essential for preserving the enzyme’s structure and proper folding. Disruption of the enzyme’s structural integrity leads to a loss of activity. The lowest chitinase activity (0.8 U/mL) was recorded at pH 11 after 48 h of incubation. Under extreme acidic or alkaline conditions, polysaccharide degradation compromises bacterial cell integrity, ultimately resulting in cell death [49]. Several studies have highlighted the critical role of medium pH in regulating chitinase production across various organisms. In the *Bacillus* sp., the maximum chitinase production has been reported at pH 8 [6]. The optimum pH for chitinase activity has been widely reported across various microorganisms. An optimal pH of 8 was observed for chitinase from *Alcaligenes xylooxydans* [23], *Serratia marcescens* XJ-01 [50], and *Streptomyces pratensis* strain KLSL55 [51]. According to Gooma et al., the optimum pH for chitinase from *Bacillus thuringiensis* and *Bacillus licheniformis* was pH 7 and 8, respectively [44]. Similarly, *Bacillus subtilis* produced chitinase most efficiently at pH 8 [52]. The highest chitinase yields were recorded at pH 7 for *Bacillus cereus* GS02 [20] and *Streptomyces chilikensis* RC1830 [53]. For *Aeromonas punctata* HS6, maximum chitinase production occurred at pH 7, while *Aeromonas hydrophila* HS4 exhibited optimal production at pH 8 [22].

### Effect of inoculum age and percentage

**Fig. 4:**
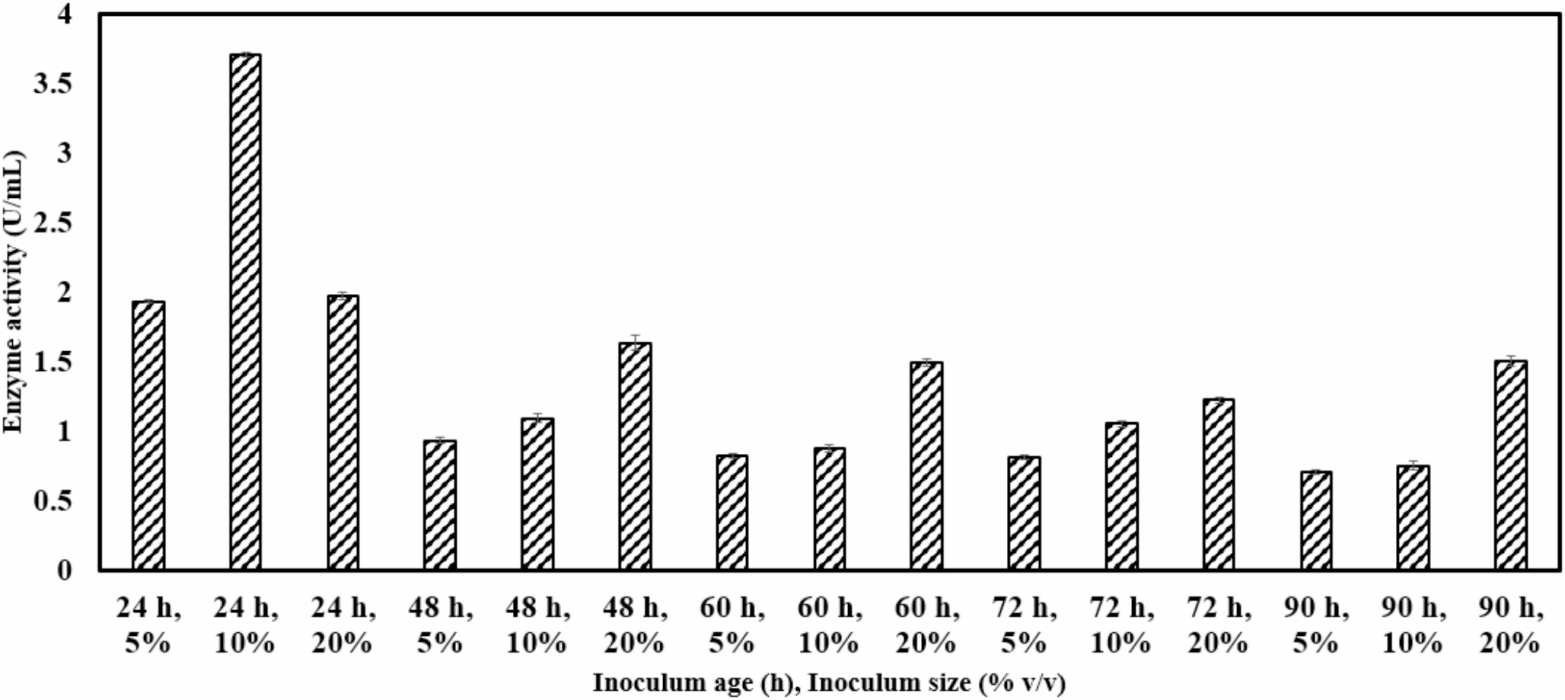
Effect of inoculum age and inoculum size on *Bacillus haynesii* chitinase activity

Optimum inoculum size is crucial for maintaining a balance between nutrient availability and proliferating biomass [54]. Chitinase activity of 1.9 U/mL was noted at 5% (v/v) inoculum size at 24 h of inoculum age. Lower microbial biomass in the production medium requires a longer time to reach an optimal cell density for efficient substrate utilization, ultimately resulting in reduced product formation [51]. In the present study, the maximum chitinase production (3.7 U/mL) was observed with an inoculum age of 24 h and an inoculum size of 10% (v/v), as shown in Fig. 4. In another study conducted, the optimal inoculum size for *Bacillus* sp. BG-11 [55], *Bacillus* sp. R2 [56], and *Cellulomonas flavigena* NTOU 1 [57] was reported to be 2%, 2.5%, and 1%, respectively. For *Enterobacter* sp. NRG4, the maximum chitinase production was achieved with an inoculum size of 2.6 mL per 4 g of solid substrate [54]. However, increasing the inoculum size to 20% (v/v) for a 24 h culture resulted in reduced chitinase activity (1.97 U/mL), possibly due to nutrient depletion, which could not support the accelerated growth rate of the culture. These results indicate that optimizing the inoculum size enhances microbial growth and enzyme production up to a threshold, beyond which growth declines due to limited nutrient availability. At 48 h of inoculum age, the chitinase activity of 0.93 U/ mL, 1.09 U/mL and 1.63 U/mL was noted for inoculum percentage of 5% (v/v), 10% (v/v) and 20% (v/v) respectively. Similarly, a study conducted with inoculum age of 60 h, 72 h and 90 h each at different inoculum percentage 5%, 10% and 20% revealed that maximum activity of 1.2 ± 0.3 U/ mL was attained at inoculum percentage of 20% (v/v). The maximum enzyme activity observed at inoculum ages of 48 h, 60 h, 72 h, and 90 h with varying inoculum sizes was comparatively lower than that of the 24 h culture. This indicates that the microbial culture reached its exponential phase at 24 h, with the highest enzyme activity of 3.7 U/mL achieved at an inoculum size of 10% (v/v). In another study conducted by Cheba et al. on *Bacillus* sp. R2 the optimum inoculum age was observed at 18 h [56]. In a similar study conducted on *Bacillus cereus* GS02 the maximum chitinase activity production was obtained with an inoculum size of 1.2% and incubation period of 48 h [20]. Hence, inoculum age of 24 h and inoculum percentage of 10% (v/v) was retained for further optimisation process as it was found to be more suitable for growth and enzyme production from *Bacillus haynesii*.

### Effect of culture volume on chitinase production

**Fig. 5:**
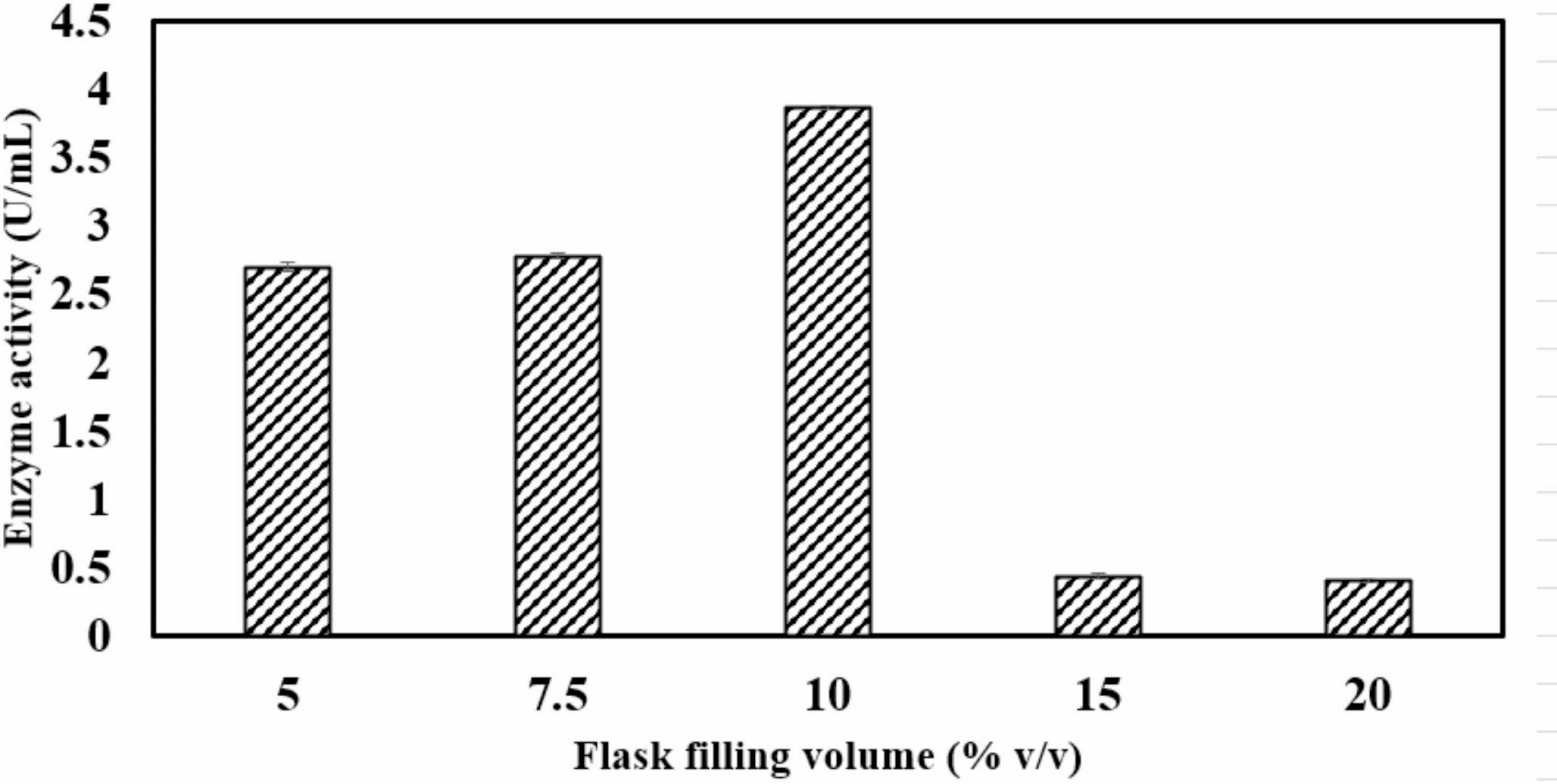
Effect of different flask filling volume on *Bacillus haynesii* chitinase activity

The 100 mL Erlenmeyer flask containing 10 mL of medium (10% v/v) demonstrated the highest chitinase activity, reaching 3.9 U/mL (Fig. 5). Flasks with medium volumes of 5 mL (5% v/v) and 7.5 mL (7.5% v/v) also supported chitinase production, suggesting that oxygen transfer remained sufficient beyond the critical threshold. However, due to nutrient limitations, the chitinase activity declined to 2.7 U/mL and 2.8 U/mL at 5% and 7.5% v/v filling volumes, respectively. A further increase in medium volume beyond 10% v/v led to a substantial reduction in chitinase production, with activity dropping to 0.4 U/mL, likely attributed to insufficient oxygen transfer rates. Cheba et al. reported a similar trend, where maximum chitinase yield was achieved at a 20% flask filling volume [56].

### Statistical optimisation: design of experiments

#### Screening of significant media components by Plackett Burman design

**Fig. 6:**
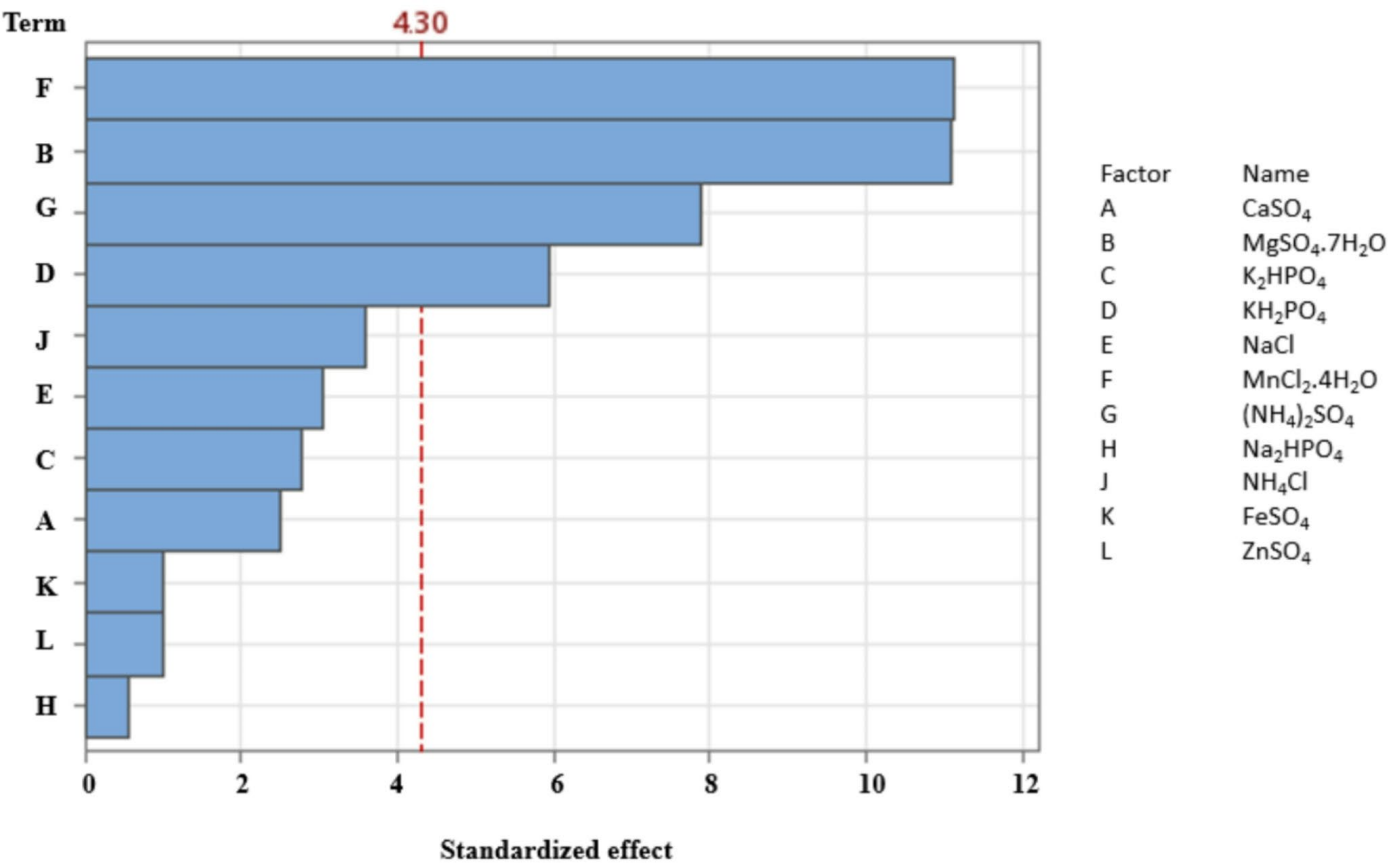
Pareto chart of independent variables with its significant effects

To investigate the impact of various parameters on chitinase activity, a Plackett–Burman experimental design was employed, resulting in 15 experimental runs as presented in Table 3. Chitinase activity, measured using the DNSA method, was analyzed statistically to identify significant factors. Parameters with a p-value < 0.05 were considered to have a substantial influence on chitinase production. The Pareto chart depicted in Fig. 6 highlights the relative impact of each parameter, with the Bonferroni limit (threshold value of 4.30) serving as a criterion for significance. Factors exceeding this limit were identified as significant contributors to chitinase activity, whereas those below the threshold were deemed insignificant. The analysis clearly demonstrated that MgSO₄·7 H₂O and MnCl₂·4 H₂O exerted the most pronounced influence on chitinase production. In contrast, KH₂PO₄ and (NH₄)₂SO₄ exhibited F-values < 2, indicating their marginal effect on chitinase activity. Consequently, the two significant parameters, MgSO₄·7 H₂O and MnCl₂·4 H₂O, were selected for further optimization using the Taguchi approach to enhance chitinase production. Similar to the obtained results, Han et al., reported that colloidal chitin and MgSO_4_.7H_2_O influence the chitinase production of *Streptomyces* sp. Da11 identified using Plackett Burman screening design [58]. Kumar et al., reported that casein, pH, chitin and NaCl has positive influence on chitinase production from *Bacillus* sp. identified using Plackett-Burman design [59]. Also, colloidal chitin, MgSO_4_ and yeast extract were represented as significant parameters for chitinase production [23–25]. Mn^2+^ was found to stimulate the chitinase production in *Paenibacillus* sp. TKU052 [60], *Serratia marcescens* B4A [48] and in *Paenibacillus chitinolyticus* strain UMBR 0002 [61]. Chitinase activity was found to increase in *Bacillus cereus* GA6 in the presence of Mg^2+^ and NH_4_Cl [62]. Interestingly, several researchers reported NH_4_Cl has significant effect on chitinase production. Therefore, we have considered NH_4_Cl for further optimisation along with other parameters using Taguchi approach.

**Table 3:** Plackett Burman Experimental design matrix with chitinase activity as response

Run	CaSO_4_	MgSO_4_	K_2_HPO_4_	KH_2_PO_4_	NaCl	MnCl_2_	(NH_4_)_2_SO_4_	Na_2_HPO4	NH_4_Cl	FeSO_4_	ZnSO_4_	Enzyme activity
	g/L	g/L	g/L	g/L	g/L	g/L	g/L	g/L	g/L	g/L	g/L	U/mL
1	3	0.6	0	0	0	0.12	0	14	2	0	0.06	4.29
2	3	0	6	12	0	0.12	2	14	0	0	0	2.51
3	0	0	0	12	0	0.12	2	0	2	0.06	0.06	4.93
4	0	0.6	0	12	10	0	2	14	2	0	0	3.58
5	0	0	6	0	10	0.12	0	14	2	0.06	0	4.20
6	0	0.6	6	0	10	0.12	2	0	0	0	0.06	3.23
7	0	0	0	0	0	0	0	0	0	0	0	0.97
8	3	0.6	0	12	10	0.12	0	0	0	0.06	0	2.33
9	3	0	6	12	10	0	0	0	2	0	0.06	2.26
10	1.5	0.3	3	6	5	0.06	1	7	1	0.03	0.03	2.84
11	0	0.6	6	12	0	0	0	14	0	0.06	0.06	2.18
12	3	0	0	0	10	0	2	14	0	0.06	0.06	0.18
13	3	0.6	6	0	0	0	2	0	2	0.06	0	3.57
14	1.5	0.3	3	6	5	0.06	1	7	1	0.03	0.03	2.84
15	1.5	0.3	3	6	5	0.06	1	7	1	0.03	0.03	2.83

#### Enhancing chitinase production by Taguchi orthogonal array approach

The major factors affecting the chitinase activity of *Bacillus haynesii* were colloidal chitin, glycerol, MgSO_4_.7H_2_O, MnCl_2_.4H_2_O and NH_4_Cl identified from One Factor at a Time (OFAT) approach and Plackett Burman design. Yeast extract, an excellent nitrogen source in production medium was also optimised in this study. Several researchers have observed that yeast extract significantly affects the chitinase production [20, 41]. Yeast extract, an organic nitrogen source is composed of complex molecules, cofactors and vitamins which play a prominent role in stimulating metabolic pathways and enzyme production [31]. Nampoothiri et al., suggested that chitin present in yeast extract can stimulate the production of chitinase [43]. Taguchi experimental design was employed to identify the optimum conditions for the production of chitinase and to enhance the yield based on signal to noise ratio. Table 4 represents the factors and its corresponding coded values. By this method, experimental variations can be eliminated providing better experimental outcome. L_25_ orthogonal array was used which resulted in 25 experimental runs. Taguchi experimental design along with its corresponding response are shown in Table 2. Response variable is the chitinase activity. The maximum chitinase activity of 6321 mU/mL was observed in run no. 19 which includes glycerol (7.5% w/v), yeast xtract (7.5 g/L), MgSO_4_. 7H_2_O (1.2 g/L), MnCl_2_. 4H_2_O (0.48 g/L) and colloidal chitin (0.6% w/v). These are the optimum combination of the parameters to obtain the maximum production of chitinase identified from larger the better S/N ratio. Larger S/N ratio is preferred as it represents optimum chitinase production and indicates the finest level for each of the parameters. The Run no. 9 containing 7.5 g/L yeast extract, 1.8% w/v colloida chitin, 0.12 g/L MnCl_2_. 4H_2_O, 2.4 g/l MgSO_4_. 7H_2_O, 2.5 g/L NH_4_Cl gave minimum activity of 213 mU/mL.

**Table 4:** Factors and coded values for Taguchi design

Factor	Name	Units	Levels	-2	-1	0	-1	-2
					Low	Center	High	+α
A	Yeast extract	g/L	5	0	2.5	5	7.5	10
B	Glycerol	% w/v	5	0	2.5	5	7.5	10
C	Colloidal chitin	% w/v	5	0d	0.6	1.2	1.8	2.4
D	MnCl_2_. 4H_2_O	g/L	5	0	0.12	0.24	0.36	0.48
E	MgSO_4_. 7H_2_O	g/L	5	0	0.6	1.2	1.8	2.4
F	NH_4_Cl	g/L	5	0	1.25	2.5	3.75	5

In the Taguchi approach, an orthogonal array design is employed to optimize the process by evaluating independent factors at specific levels. This method enables the identification of optimal process conditions, the assessment of factor contributions, and the prediction of responses under these optimized conditions [32]. The Taguchi approach requires a minimal number of experimental runs to determine optimal conditions. The size of the experiment is a critical factor, as it directly impacts cost and time. The primary advantage of the Taguchi design over Response Surface Methodology (RSM) lies in its ability to identify key factors and achieve optimized conditions with fewer experiments [29, 32]. Few researchers have investigated the application of the Taguchi approach for optimizing chitinase production. Zarei et al., utilized this method to optimize chitinase production from *Serratia marcescens* B4A [32] whereas Aliabadi et al., optimised chitinase production from *Cohnella* sp. A01 by Taguchi approach [30]. Atheena et al. investigated chitinase production from Bacillus thuringiensis and optimized the process using the Taguchi design, achieving a chitinase yield of 13.46 U/mL [31].

**Fig. 7:**
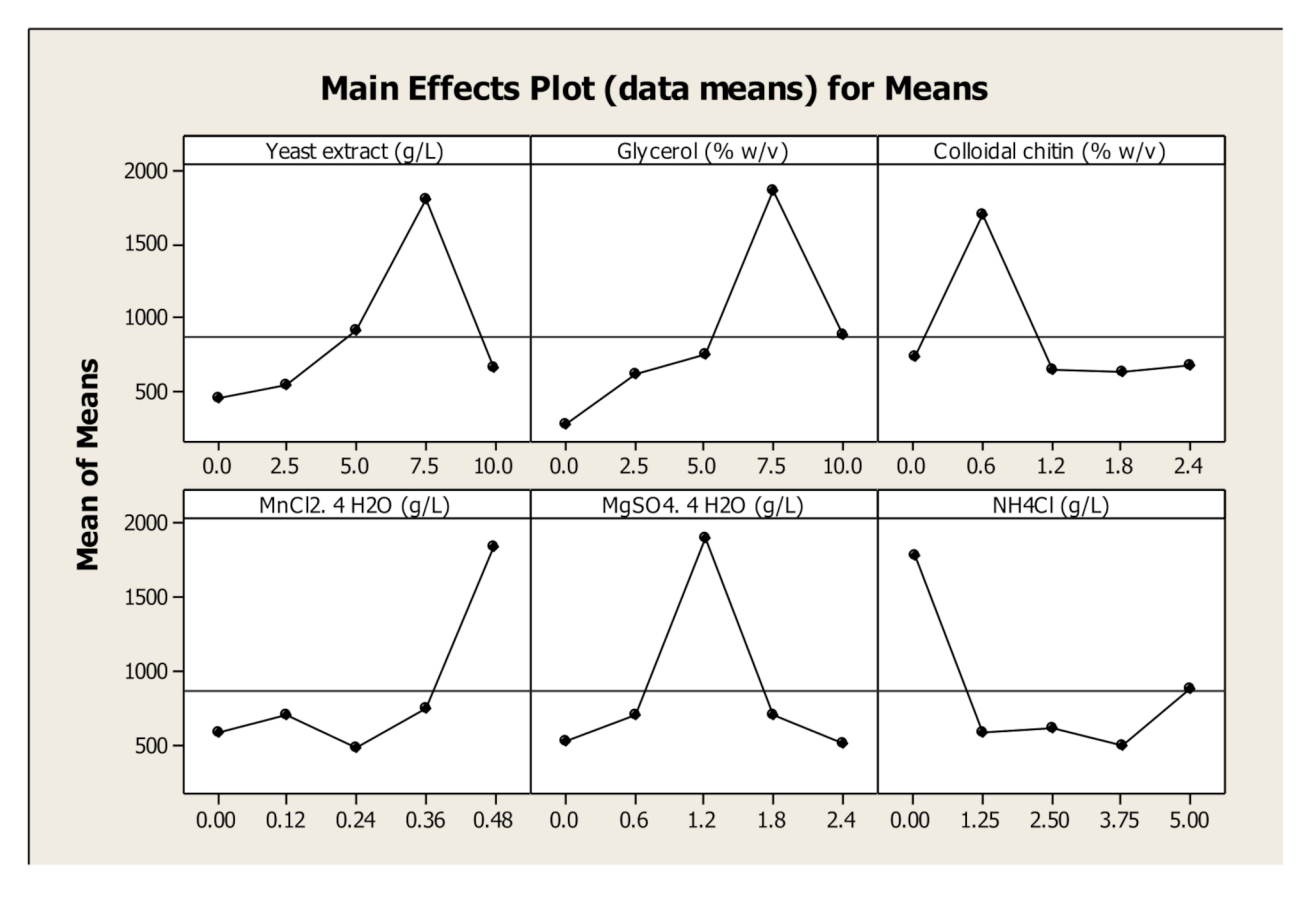
Main effects plot for all individual parameters on chitinase yield from *Bacillus haynesii*

The main effects of 6 factors at five levels on chitinase enzyme generated using MINITAB software is represented in Fig. 7. This plot represents the changes in response with respect to the given factor from low level to high level.

**Fig. 8:**
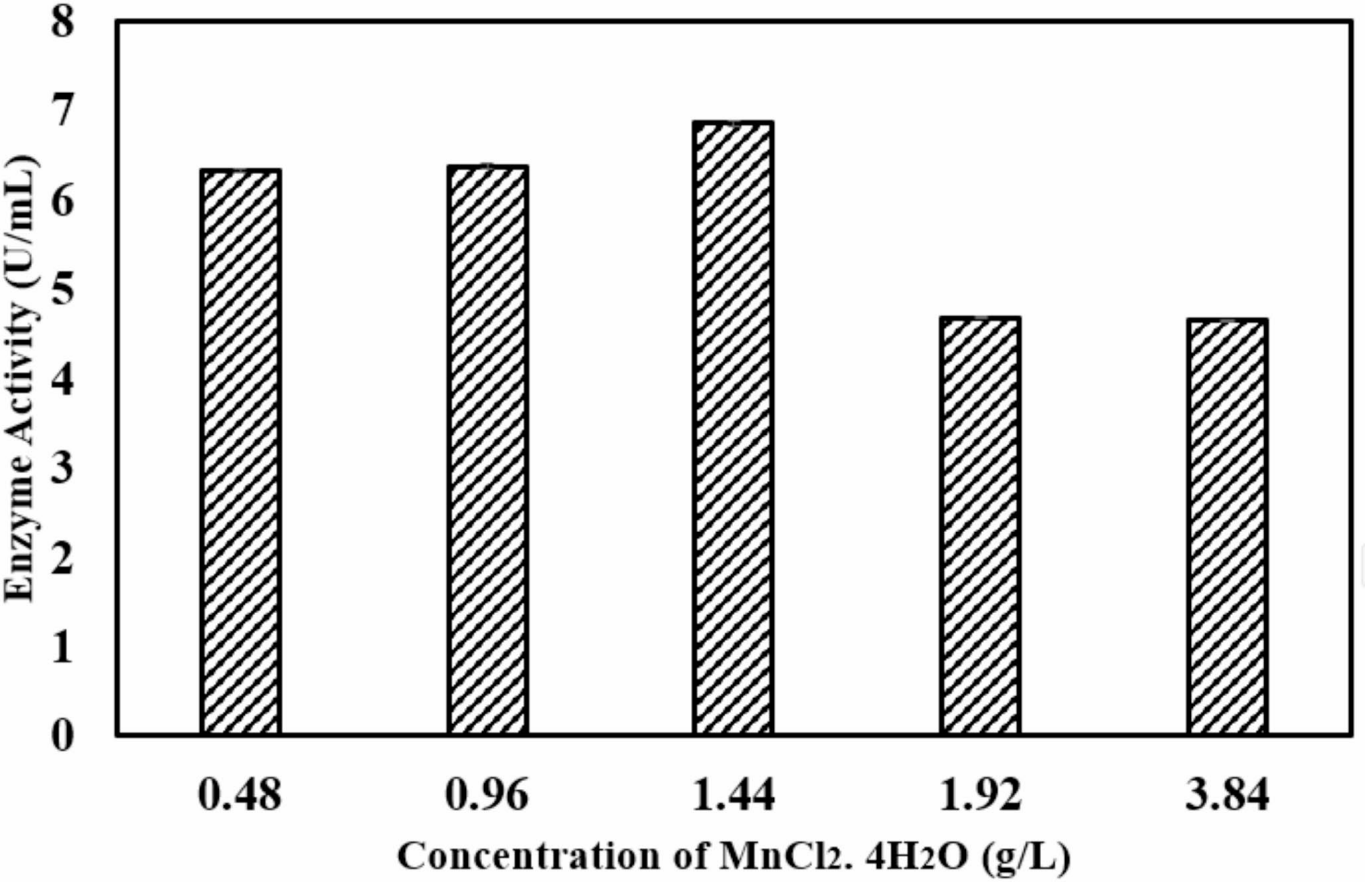
Augmented Taguchi design of MnCl_2_.4H_2_O by One Factor at a Time (OFAT) approach

In this plot, response is plotted against each parameter level. If the line is horizontal to X-axis, the main effect of the parameter is negligible whereas if the line shows larger deviation from X- axis, the main effect of the parameter is maximum [63]. From this plot, the optimal parameters and their corresponding levels were identified. It was concluded that NH₄Cl at level 1, colloidal chitin at level 2, MgSO₄·7 H₂O at level 3, yeast extract and glycerol at level 4, and MnCl₂·4 H₂O at level 5 had the maximum impact on chitinase yield. All parameters reached their highest point within the tested range, except MnCl₂·4 H₂O. This indicates that excessive amounts of MgSO₄·7 H₂O, colloidal chitin, yeast extract, and glycerol had an adverse effect on chitinase activity. MnCl₂·4 H₂O showed a significant increase in activity beyond the tested range. Therefore, the effect of MnCl₂·4 H₂O on chitinase activity was further analyzed, as shown in Fig. 8. NH₄Cl had no measurable effect on chitinase yield.

Further, experimental variations caused by uncontrollable parameters can be avoided with the help of S/N ratio. Average S/N ratio of each factor is represented in Table 5. In Taguchi approach, parameters are categorized into “Signal” factors and “Noise” factors where signal factors lead the experimental course and noise factors bring in the variability. Generally, signal factors are important to identify the deviation between response and the affecting factors. S/N ratio denotes which confluence of parameters has the maximum effect on chitinase production. Higher S/N ratio represents better performance of the specific parameter on chitinase production [31, 63]. S/N ratios were calculated for each factor and level as represented in Table 5. The degree of effect for each parameter is represented by delta, with the parameter ranked 1 exhibiting the highest delta value. Glycerol showed the greatest contribution, ranking 1 for delta, followed by MnCl₂·4 H₂O, yeast extract, and MgSO₄·7 H₂O, as summarized in Table 5.

**Table 5:** Response table for signal to noise ratio

Level	Yeast extract	Glycerol	Colloidal chitin	MnCl_2_.4H_2_O	MgSO_4_. 7H_2_O	NH_4_Cl
1	51.23	47.95	55.38	53.43	54.28	58.24
2	54.32	54.78	57.39	55.10	55.00	54.51
3	58.59	56.24	55.27	52.92	58.71	54.27
4	58.85	60.84	55.05	56.92	56.65	53.55
5	55.57	58.77	55.47	60.61	53.91	57.99
Delta	7.62	12.87	2.35	7.69	4.81	4.69
Rank	3	1	6	2	4	5

ANOVA (Table 6) highlights the significance of each parameter based on the mean square values. The coefficient of determination (R² = 0.975) indicates that 97.5% of the response variability is explained by the model. All parameters showed a significant effect on chitinase production, except MnCl₂·4 H₂O (*p* > 0.05). The percentage contribution of each factor was calculated by dividing its sum of squares by the total sum of squares and multiplying by 100. The results revealed that glycerol and colloidal chitin made the highest contributions, accounting for 44.3% and 18.6%, respectively, to chitinase production from *Bacillus haynesii.* Maximum chitinase activity was observed when the concentration of colloidal chitin in the production medium was 0.6% (w/v). An increase in colloidal chitin concentration from 1.2% (w/v) to 2.4% (w/v) resulted in a decline in enzyme activity, likely due to substrate inhibition. A similar trend was reported for *Streptomyces pratensis* strain KLSL55, where the highest chitinase activity of 157.14 IU was recorded at 1.5% colloidal chitin, which then dropped to 19.29 IU when the concentration increased to 2.5% [51]. In another study conducted on *Bacillus* sp. 13.26 the optimum chitin concentration in medium was found to be 0.5% [39].ANOVA (Table 6) illustrates the importance of parameters in comparison with mean square. R^2^ = 0.975 showed that the response variability of 97.5% could be explained by the model. All parameters showed significant effect except MnCl_2_. 4H_2_O (*p* > 0.05). The percentage contribution for each factor was calculated as sum of squares to the total sum of squares and multiplying by 100. Results revealed that glycerol and colloidal chitin had maximum contribution of 44.3% and 1.6% respetively on chitinase production from *Bacillus haynesii*. Highest chitinase activity was recorded when colloidal chitin concentration in production medium was 0.6% (w/v) Further increase in colloidal chitin concentration from 1.2% (w/v)to 2.4% (w/v)resulted in reduced enzyme activity due to substrate inhibition. Similar observations were seen in *Streptomyces pratensis* strain KLSL55 where highest chitinase activity of 157.14 IU was recorded at 1.5% of coloidal chitin beyond which the activity was reduced to 19.29 IU at 2.5% of colloidal chitin [51]. Optimum chitin concentration in medium was 0.5% for the production of chitinase from *Bacillus* sp. 13.26 [39].

**Table 6:** Analysis of Variance (ANOVA) for Taguchi model

Source	Sum of Squares	df	Mean square	F-value	*p*-value		Percentage contribution
Model	11421.07	20	571.05	10.32	0.0177	Significant	
A: Yeast extract	1429.19	4	357.30	6.46	0.0491		12.27
B: Glycerol	5158.38	4	1289.60	23.31	0.0049		44.3
C: Colloidal chitin	2168.29	4	542.07	9.80	0.0241		18.6
D: MnCl_2_. 4H_2_O	1072.24	4	268.06	4.85	0.0778		9.2
E: MgSO_4_. 7H_2_O	1592.96	4	398.24	7.20	0.0410		13.6
Residual	221.27	4	55.32				
Cor Total	11642.33	24					

R^2^ = 0.975; *df*– Degree of freedom

#### OFAT approach for augmentation of Taguchi design

Optimization using the Taguchi approach led to the media formulation consisting of glycerol (7.5% w/v), yeast extract (7.5 g/L), MgSO₄·7 H₂O (1.2 g/L), and colloidal chitin (0.6% w/v). Given that MnCl₂·4 H₂O exhibited a significant increase in chitinase activity beyond the tested range, its concentration (0.48 g/L–3.84 g/L) was further optimized using the One-Factor-at-a-Time (OFAT) method, with all other parameters kept constant. An increment of MnCl₂·4 H₂O concentration to 1.44 g/L resulted in a chitinase activity of 6.85 U/mL, with a specific activity of 28.87 U/mg. In another study performed on *Bacillus* sp. R2 supplementation with 1 mM Mn²⁺ resulted in a fourfold increase in chitinase activity [64]. In *Melghiribacillus thermohalophilus*, incorporation of 2 mM Mn^2+^ also led to an enhanced relative chitinase activity of 127% [65].

#### Ethanol production from chitin oligosaccharides using *Saccharomyces cerevisiae*

To investigate maximum ethanol production by *Saccharomyces cerevisiae*, the fermentation was carried out using 33.18 g/L of chitin oligosaccharides derived from the broth supernatant of a 48-h *Bacillus haynesii* culture. Several studies have reported the production of chitin oligosaccharides. *Chitinolyticbacter meiyuanensis* SYBC-H1 produced 2.65 g/L of N-acetylglucosamine (NAG) from chitin powder [66]. Similarly, Trichoderma harzianum AUMC 5408 yielded 12.785 ± 0.77 g/L of NAG from colloidal chitin [3]. In the present study, 2.48% v/v ethanol was produced from 33.18 g/L of chitin oligosaccharides, yielding a coefficient of 0.74.

The incorporation of chitin substrates for ethanol production has been explored by many authors. In a study conducted on *M. circinelloides* NBRC 4572 and *M. ambiguous* NBRC 8092 ethanol production of 14.5 ± 0.2 g/L and 16.9 ± 0.2 g/L were obtained respectively using NAG as substrate [67]. Xu et al., reported production of ethanol with the yield of 7.72 mL/100 g of chitin substrate using *Pleurotus ostreatus* as the organism for fermentation [68]. In another study, genetically engineered *Saccharomyces cerevisiae* was employed to produce 3 g/L ethanol using NAG as substrate [69]. In another study on *S. stipites* NBRC1687, 10,007 and 10,063 strains, 50 g/L of NAG yielded in a the ethanol production of 14.5 ± 0.6, 15.0 ± 0.3 and 16.4 ± 0.3 g/L respectively [70]. In another study, insect waste, a chitin source was used as substrate for production of 11.9 g/L of ethanol using *M. circinelloides* (AUMC 6017) [3]. In another study, 7.4 g/L of ethanol was produced from 30 g/L chitin oligosaccharides with an ethanol yield of 0.25 g of ethanol/g substrate using *Mucor circinelloides* [1]. In a study conducted on *Z. mobilis* 598 µg/µL of ethanol was produced with 10 mg/mL glucosamine as substrate [71].

## Conclusion

Amid the growing demand for fossil fuels, bioethanol emerges as a clean alternative that helps mitigate global warming. In this study, the production of chitinase from *Bacillus haynesii* was optimized using the Plackett-Burman design followed by the Taguchi orthogonal array approach, resulting in an enzyme activity of 6.85 U/mL. Subsequently, chitin oligosaccharides were utilized as a substrate for bioethanol production by *Saccharomyces cerevisiae*, with ethanol concentration measured using the potassium dichromate oxidation assay. A bioethanol concentration of 2.48% v/v was produced from 33.18 g/L chitin oligosaccharides. The findings suggest that chitin oligosaccharides are an effective substrate for bioethanol production. The process parameters can be further optimized through synthetic biology approaches to enhance the yield even further.

## Data Availability

Not applicable.
